# Systemic Inflammation Accelerates Changes in Microglial and Synaptic Markers in an Experimental Model of Chronic Neurodegeneration

**DOI:** 10.3389/fnins.2021.760721

**Published:** 2022-01-04

**Authors:** Joe K. Chouhan, Ursula Püntener, Steven G. Booth, Jessica L. Teeling

**Affiliations:** Biological Sciences, Faculty of Environmental and Life Sciences, University of Southampton, Southampton, United Kingdom

**Keywords:** neurodegeneration, microglia, synapses, bacterial infection, systemic inflammation

## Abstract

Bacterial infections are a common cause of morbidity and mortality in the elderly, and particularly in individuals with a neurodegenerative disease. Experimental models of neurodegeneration have shown that LPS-induced systemic inflammation increases neuronal damage, a process thought to be mediated by activation of “primed” microglia. The effects of a real systemic bacterial infection on the innate immune cells in the brain and neuronal networks are less well described, and therefore, in this study we use the ME7 prion model to investigate the alterations in microglia activation and phenotype and synaptic markers in response to a low grade, live bacterial infection. Mice with or without a pre-existing ME7 prion-induced neurodegenerative disease were given a single systemic injection of live *Salmonella typhimurium* at early or mid-stage of disease progression. Immune activation markers CD11b and MHCII and pro-inflammatory cytokines were analyzed 4 weeks post-infection. Systemic infection with *S. typhimurium* resulted in an exaggerated inflammatory response when compared to ME7 prion mice treated with saline. These changes to inflammatory markers were most pronounced at mid-stage disease. Analysis of synaptic markers in ME7 prion mice revealed a significant reduction of genes that are associated with early response in synaptic plasticity, extracellular matrix structure and post-synaptic density, but no further reduction following systemic infection. In contrast, analysis of activity-related neuronal receptors involved in development of learning and memory, such as *Grm1* and *Grin2a*, showed a significant decrease in response to systemic bacterial challenge. These changes were observed early in the disease progression and associated with reduced burrowing activity. The exaggerated innate immune activation and altered expression of genes linked to synaptic plasticity may contribute to the onset and/or progression of neurodegeneration.

## Introduction

Alzheimer’s disease (AD) is the most common form of dementia, accounting for approximately 80% of all dementia cases (Anand et al., 2014). Sporadic AD, or late-onset AD, is characterized by progressive memory loss and a reduction in higher cognitive functions, due to loss of synapses, extracellular deposits of amyloid beta, hyperphosphorylation of tau and neuroinflammation (Anand et al., 2014). Systemic inflammation also plays a role in the onset and/or progression of cognitive decline. Serum levels of inflammatory cytokines are significantly increased in people with dementia compared to healthy controls (Stoeck et al., 2014; Dursun et al., 2015; Ott et al., 2018) and clinical studies show that low-grade systemic inflammation, for example following a bacterial or viral infection, can modify neuropathology and this is linked to a faster rate of cognitive decline (Holmes et al., 2003; Heneka et al., 2015; Ide et al., 2016; Rakic et al., 2018). We, and others have shown that serum TNF-α levels are increased in people with AD, which contributes to faster disease progression and inhibition using the TNF-α inhibitor, etanercept, showed a trend toward a slower rate of decline (Holmes et al., 2009, 2011; Butchart et al., 2015). Microbial infections have also been studied in experimental models of neurodegeneration (McManus et al., 2014; Singer et al., 2016; Ding et al., 2018; Ilievski et al., 2018; Dominy et al., 2019; Basak et al., 2021). These studies show that systemic microbial infections or bacterial sepsis can lead to enhanced amyloid load, neuroinflammation and cognitive impairment in transgenic models of AD, but the effect on a real live, low-grade bacterial infection in a prion model has not yet been studied.

The murine ME7 prion model of chronic neurodegeneration is an experimental mouse model where both central and systemic inflammation impact on disease progression (Chouhan et al., 2017). The murine ME7 prion model is mouse-adapted form of transmissible spongiform encephalopathy that shares characteristic hallmarks of human neurodegenerative diseases, including vacuolation of the gray matter, reactive gliosis, synaptic loss, and death of hippocampal pyramidal cells resulting from deposition of misfolded prion protein (PrP^Sc^) (Chouhan et al., 2017). Previous experiments investigating the effects of systemic inflammatory challenge in the murine ME7 prion model have shown that administration of the bacterial mimetic, lipopolysaccharide (LPS), induces acute changes in behavior. These changes in behavior are associated with an increase in microglial activation and cytokine production in disease-affected brain regions and exacerbation of disease pathology (Cunningham et al., 2009; Field et al., 2010; Lunnon et al., 2011; Murray et al., 2011; Hennessy et al., 2015; Skelly et al., 2019). In ME7 prion-inoculated mice, microglial activation is observed from 8 weeks post-inoculation (wpi) (Betmouni et al., 1996), while the first changes to neuronal networks, including synaptic loss, are detected from 12-wpi (Chiti et al., 2006; Gray et al., 2009; Hilton et al., 2013). Many studies investigating the effect of systemic inflammation on neurodegeneration have used mimetics of infection as opposed to live infection and the effects on synaptic markers are less well described.

Systemic bacterial infection can be modeled in mice using an attenuated strain of the Gram-negative bacterium, *Salmonella typhimurium* (*S. typhimurium* SL3261) (Peters et al., 2010; Puntener et al., 2012). In cognitively naïve mice, infection with *S. typhimurium* SL3261 results in prolonged activation of the cerebral vasculature and induction of cytokine expression 3 weeks after infection and therefore provides a valuable model for low-grade, chronic systemic inflammation (Puntener et al., 2012). The changes to microglial activation and synaptic expression levels following a real bacterial infection remain unknown. Investigating how a real, live bacterial infection alters the neuroinflammatory status in a model of neurodegeneration would increase our understanding of the mechanisms that are involved in the effects of chronic systemic inflammation, and possibly the progression of AD. Our results suggest that systemic inflammation in the early stages of ME7 prion disease exacerbates neuroinflammation and synaptic pathology. These observations may help to understand the hastened cognitive decline observed in patients with chronic neurodegenerative disease.

## Materials and Methods

### Experimental Animals

All mice detailed in the following experiments were of the strain C57BL/6. Male only or female only mice were used in different experiment. Mice (8–12 weeks old) were housed in groups of four to six, under a 12-h/12-h light-dark cycle at 19–23°C, with water and normal chow diet (RM1, SDS, United Kingdom) *ad libitum*. All mice were maintained at the Biomedical Research Facility, Southampton General Hospital, Southampton, United Kingdom.

### Experimental Model of Prion Disease

Brain homogenate (10% w/v) from normal mice (NBH) or from terminal ME7 prion mice (ME7) was directly injected into the dorsal hippocampi of mice using a stereotaxic frame. Female C57BL/6 mice (10–12 weeks) were anesthetized with a ketamine/rompun mixture (85 mg/kg and 13 mg/kg), and the incision area was shaved with hair clippers (Wella, United Kingdom) and sterilized with 70% alcohol. Lacri-lube (Allergan, United Kingdom) was applied to each eye to prevent drying that comes from the removal of the blink reflex under anesthesia. The mouse was fitted to a stereotactic frame (Kopf Instruments, United States) using 45° atraumatic ear bars and an incisor bar (Kopf Instruments, United States). A sagittal incision was made exposing the skull, allowing for the location of bregma and burr holes were drilled bilaterally into the skull using a dentist’s drill, taking care not to damage the dura mater, at the appropriate coordinates: 2.0 mm posterior to bregma and ± 1.7 mm lateral to the midline. A 10 μl syringe with a 26s-gauge needle (Hamilton, United Kingdom) was inserted into the brain to a depth of 1.6 mm, and 1 μl of brain homogenate was injected at a flow rate of 1 μl/min. The needle remained in place for 2 min to allow for bolus diffusion, before being slowly removed. The incision was closed with sutures before the mice were placed in a heated chamber to recover. After recovery, mouse appearance was regularly checked over the following week, and weights were measured weekly thereafter, to ensure animal welfare was maintained.

### Burrowing

Plastic cylinders, 20 cm long and 6.8 cm in diameter, were filled with 190 grams of normal chow food pellets (RM1, SDS) and placed in the cages. Mice were habituated once with a full tube in a group cage and then twice in individual cages with a full burrowing tube to establish baseline before weekly testing. For testing, mice were placed individually in cages overnight and the remaining pellets at the end of each session were weighed, with the amount displaced (“burrowed”) recorded. The mice were returned to their home cage after testing. Relative weight of pellets burrowed across successive weeks was compared to initial baseline and presented as percentage of baseline.

### Infection With Salmonella Typhimurium

An attenuated strain of *Salmonella typhimurium*, *S. typhimurium* SL3261, has been used previously to model low-grade systemic inflammation and shows changes in both peripheral and central cytokine expression (Peters et al., 2010; Puntener et al., 2012). ME7 prion mice were given an intraperitoneal (i.p.) injection of 1 × 10^6^ colony forming units (cfu) *S. typhimurium* SL3261 or 200μl non-pyrogenic saline (Kent Pharmaceuticals, United Kingdom) at either 8- or 12-weeks post-inoculation (wpi). NBH-injected control mice were given a single i.p. injection of 200 μl non-pyrogenic saline or *S. typhimurium* (ME7 + SL3261). Mice were transcardially perfused with 0.9% saline containing heparin sodium (5 U/ml; CP Pharmaceuticals, United Kingdom), 4 weeks after exposure to *S. typhimurium* SL3261 (12 wpi or 16 wpi). Tissue was collected for immunohistochemistry and biochemical analysis of microglial activation and synaptic marker gene expression.

### Immunohistochemistry

Immunohistochemistry was carried out on 10 μm-thick fresh frozen tissue sections cut from embedded brains using a Leica CM3050 cryostat. Tissue sections were air dried, fixed in cold ethanol, quenched with 1% hydrogen peroxide (Sigma-Aldrich, United Kingdom) in phosphate-buffered saline (PBS) and blocked with 2% (w/v) bovine serum albumin (Thermo Fisher Scientific, United Kingdom), 10% (v/v) normal rabbit serum (Sigma-Aldrich, United Kingdom) in PBS at room temperature (RT) for 30 min. Primary antibodies for rat anti-mouse major histocompatibility complex class II (MHCII; 14-5321-82, Thermo Fisher Scientific, United Kingdom) and rat anti-mouse CD11b (MA5-16258, Thermo Fisher Scientific, United Kingdom) were diluted 1:500 in PBS and incubated overnight at 4 degrees. Following incubation with the primary antibody, sections were washed and incubated with biotinylated rabbit anti-rat IgG antibody (BA-4000; Vector Laboratories, United Kingdom) for 60 min at RT. Sections were washed and incubated with Vectastain ABC kit (PK-4000; Vector Laboratories, United Kingdom) for 30 min at RT and staining developed with 0.05% (v/v) diaminobenzidine (DAB; Sigma Aldrich, United Kingdom) in phosphate buffer with 0.015% (v/v) hydrogen peroxide. Sections were counterstained with Harris hematoxylin (Sigma-Aldrich, United Kingdom) and differentiated in acid alcohol, dehydrated in increasing ethanol concentrations and xylene before mounting with DPX (Thermo Fisher Scientific, United Kingdom).

### Quantification of Immunohistochemistry Images

Images were captured using a Leica DM5000B microscope and a DFC300 FX camera at 2.5 × optical zoom with LAS X software (Leica Microsystems). All brightfield images were captured using identical exposure limit, gain and saturation. DAB staining was separated from other channels with the Color Deconvolution plugin using FIJI, built on ImageJ v2 (Ruifrok and Johnston, 2001; Rueden et al., 2017). Image threshold limit for each marker was set to a level removing background noise and was kept constant for all images. Images were converted to a binary image and percentage area of staining was quantified: the fold increase in area covered was calculated relative to ME7 + saline animals at the respective time point. At least every 4th section was taken for analysis to reduce the likelihood of counting a cell twice and area used to avoid over-valuing cell fragments.

### Analysis of mRNA Transcripts by qPCR

Tissue samples enriched for the hippocampus and thalamus were isolated following transcardial perfusion and snap-frozen in liquid nitrogen. For mRNA analysis, samples were homogenized in 1 ml TRIzol reagent (Thermo Fisher Scientific, United Kingdom) using a motor-driven pellet pestle. After a 5-min incubation at RT, 200 μl 1-bromo-3-chloropropane (Sigma Aldrich, United Kingdom) was added, the mixture shaken vigorously for 15 s and incubated for 3 min at RT followed by centrifugation at 12,000 × *g* for 15 min at 4 degrees. 300 μl of the resultant RNA-containing aqueous phase was added to 500 μl RNase-free isopropanol (Sigma Aldrich, United Kingdom) and the samples incubated at room temperature for 10 min following a vigorous mixing. Samples were centrifuged at 12,000 × *g* for 10 min at 4 degrees before excess isopropanol removed and the pellet washed with 1 ml RNase-free ethanol (70% v/v). Samples were centrifuged at 7,000 × *g* for 10 min at 4 degrees before ethanol removed and samples air-dried at RT for 15 min. RNA was eluted in 20 μl. DNase/RNase-free water (Life Technologies, United Kingdom) before being placed at 60 degrees for 10 min. Samples were spun down before contaminants were removed using RNeasy MinElute Cleanup Kit (Qiagen, United Kingdom) as per manufacturers’ instructions.

Isolated RNA was quantified using a NanoDrop ND-1000 and all samples with an A_260_:A_280_ ratio of ≥ 1.8 were included. Four hundred nanogram of sample RNA was retrotranscribed using the TaqMan Reverse Transcription Reagents kit (Thermo Fisher Scientific, United Kingdom) following the manufacturer’s instructions for cDNA synthesis with random hexamers. qPCR analysis was performed in duplicate using 1x iTaq Universal SYBR Green Supermix (Bio-Rad, United Kingdom), as previously described (Ibbett et al., 2019). Primers, detailed in Table 1, were designed using Primer-BLAST (NCBI) with one primer of each pair recognizing an exon-exon boundary to prevent amplification of genomic DNA (Supplementary Table 1). GAPDH was used as a reference gene and relative expression of target genes calculated using the following equation based on the threshold cycle (Ct) values: 2^(GAPDH Ct–target gene Ct)^. Fold change in expression between treatment groups was calculated relative to target gene expression in ME7 + saline mice.

**TABLE 1:** SYBR green qPCR primer sequences.

Gene	Protein name	Strand	Primer sequence (5′–3′)
*Gapdh*	Glyceraldehyde 3-phosphate dehydrogenase	Forward Reverse	TCCACCACCCTGTTGCTGTA TGAACGGGAAGCTCACTGG
*Grin2a*	NMDA receptor subunit 2a	Forward Reverse	TTGTCTCTGCCATTGCTGTC ATATGGCTCCTCTGGGGCCT
*Grm1*	Metabotropic glutamate receptor 1 (mGluR1)	Forward Reverse	AGGCAAGGGCGATGCTTGAT AGCATCCATTCCACTCTCGC
*Il1b*	Interleukin 1-beta (IL-1β)	Forward Reverse	CAAAAGATGAAGGGCTGCTTCC ATGTGCTGCTGCGAGATTTG
*Syp*	Synaptophysin	Forward Reverse	GAGAACAACAAAGGGCCAAT GCACATAGGCATCTCCTTGA
*Tnf*	Tumor necrosis factor alpha (TNF-α)	Forward Reverse	CGAGGACAGCAAGGGACTA GCCACAAGCAGGAATGAGAA

### PCR Profiler Array

Isolated RNA from ME7 + saline and ME7 + SL3261 brain samples taken at 12 wpi was pooled (*n* = 5 per treatment group) and analysis of synaptic plasticity genes was conducted in using an RT^2^ Profiler PCR Array Mouse Synaptic Plasticity (330231; QIAGEN, United Kingdom). Sample preparation and loading was carried out as detailed in the manufacturer’s instructions. Analysis was completed using the provided analysis software.

### Statistical Analysis

Prism software (v8.4.3; Graphpad, United States) was used to perform statistical analyses and graphically present data as mean ± SD, along with individual data points. All data were subject to testing for normal and lognormal distribution using the Shapiro-Wilk test. If data was normally distributed, data were then analyzed with the appropriate parametric test and *post hoc* analysis depending on experimental design and number of independent variables, as described in the figure legend. If data failed to meet normal distribution but passed lognormality test, data were log transformed and analyzed with a parametric test. If normality was not met with log transformation, data were analyzed using the appropriate non-parametric equivalent statistical test as detailed in the figure legends.

## Results

Our previous study using *S. typhimurium* in wild-type mice identified changes in microglial activation, cytokine expression, vascular activation, and splenomegaly (Puntener et al., 2012). The effect of systemic bacterial infection with *S. typhimurium* on the central immune response of ME7 mice was thus investigated by changes in microglial activation markers and cytokine expression. Mice infected with *S. typhimurium* showed the expected changes in body weight following infection measured over 4 weeks after bacterial infection (Supplementary Figure 1).

### Systemic Bacterial Infection in ME7 Prion Mice Induces Enhanced and Prolonged Microglial Activation in the Hippocampus and Thalamus

**FIGURE 1:**
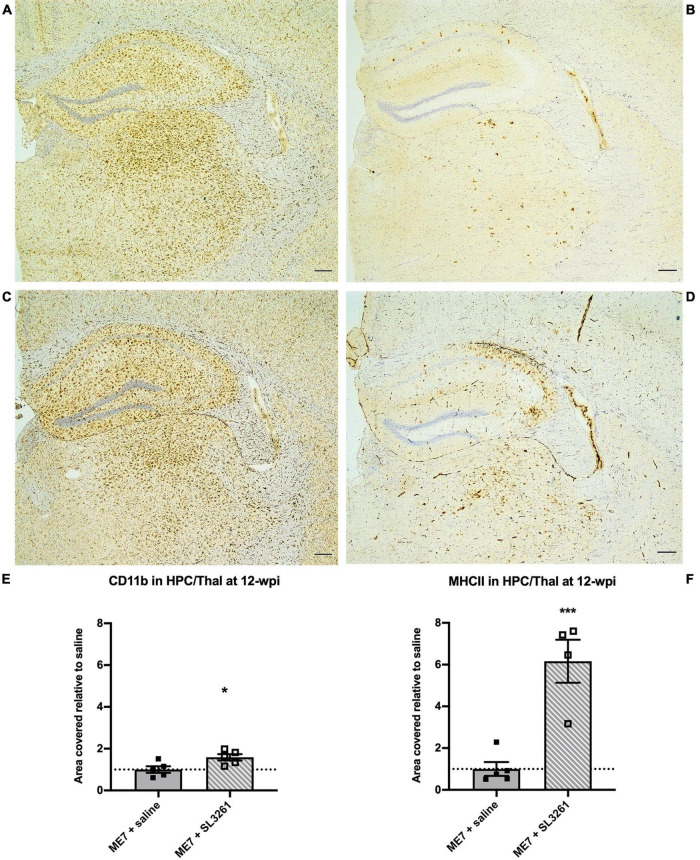
Neuroinflammatory response at early stage ME7 prion disease. Neuroinflammatory response in the hippocampus and thalamus of ME7 prion mice 4 weeks after systemic injection of saline or 1 × 10^6^ cfu *S. typhimurium* SL3261 at 8-wpi. Representative images showing CD11b **(A,C)** and MHCII **(B,D)** expression. Scale bar = 200 microns. Quantification of staining shows increased expression after systemic infection with *S. typhimurium* compared to saline injection for CD11b **(E)** and MHCII **(F)**. *p < 0.05; ***p < 0.001 vs. ME7 + saline using one-tailed unpaired Students’ *t*-test on log-transformed values; *n* = 4–5/group.

To investigate the effect of a real, live bacterial infection at the early stage of disease, ME7 prion mice were infected with 1 × 10^6^ cfu *S. typhimurium* SL3261 (ME7 + SL3261) or saline and phenotypic changes to microglia were measured 4 weeks later (12-wpi). Microglia in the hippocampus and thalamus showed significant changes in expression of CD11b and MHCII after *S. typhimurium* infection (Figure 1). CD11b expression was significantly increased compared to saline-injected ME7 prion mice [*t*(8) = 2.683, *p* = 0.0139] (Figures 1A,C,E). Increased expression of MHCII is evident on cells that resemble microglia, but also on cells that resemble blood vessels, following infection with *S. typhimurium* [*t*(7) = 5.397, *p* = 0.0005] (Figures 1B,D,F). The increased expression of these immune activation markers indicates an exaggerated neuroinflammatory response.

**FIGURE 2:**
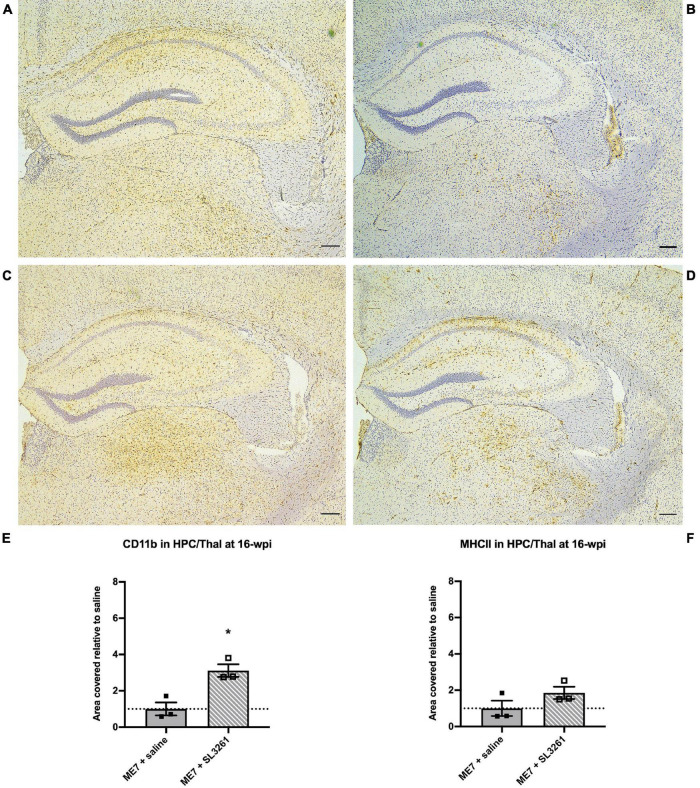
Neuroinflammatory response at mid-stage ME7 prion disease. Neuroinflammatory response in the hippocampus and thalamus of ME7 prion mice 4 weeks after systemic injection of saline or 1 × 10^6^ cfu *S. typhimurium* SL3261 at 12-wpi. Representative images showing CD11b **(A,C)** and MHCII **(B,D)** expression. Scale bar = 200 microns. Quantification of staining shows increased expression after systemic infection with *S. typhimurium* compared to saline injection for CD11b **(E)** but not MHCII **(F)**. *p < 0.05 vs. ME7 + saline using one-tailed Mann-Whitney *U*-test; *n* = 3/group.

Next, we investigated the effects of *S. typhimurium* infection in ME7 prion mice at mid-stage of disease. Analysis of the immune activation markers CD11b and MHCII, analyzed 4 weeks later (16-wpi), showed changes in expression levels (Figure 2). Increased CD11b expression on microglia [*U*(6, 15) = 0, *p* = 0.05] was observed in the hippocampus and thalamus after *S. typhimurium* infection (Figures 2A,C,E). Although MHCII expression appeared increased (Figures 2B,D,F), expression was not statistically different 4 weeks after *S. typhimurium* infection compared to saline-injected mice [*U*(8, 13) = 2, *p* = 0.20].

### Functional Changes in Microglia Following Systemic Bacterial Infection Are Dependent on Underlying Disease Pathology in ME7 Prion Mice

**FIGURE 3:**
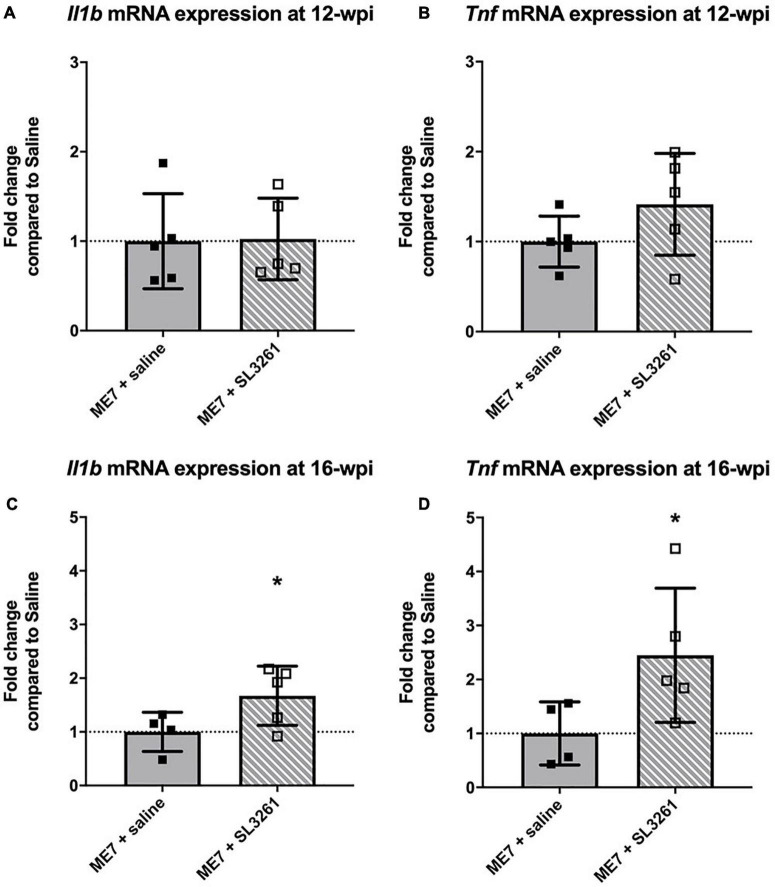
Cytokine gene transcription. The impact of systemic bacterial infection on central cytokine gene transcription in ME7 prion mice at 12-wpi **(A,B)** and at 16-wpi **(C,D)**, 4 weeks after injection of saline or *S. typhimurium* SL3261 (10^6^ cfu, i.p.). mRNA expression of inflammatory cytokines IL-1β **(A,C)** and TNF-α **(B,D)** was increased following infection in ME7 prion mice at 16-wpi, but not 12-wpi. *p < 0.05 using one-tailed unpaired Students’ *t*-test; *n* = 4–5/group.

To determine if microglia underwent a functional change following systemic infection with *S. typhimurium*, we measured the expression of the proinflammatory cytokines, IL-1β and TNF-α (Figure 3). Expression levels of IL-1β [*t*(8) = 0.8024, *p* = 0.469] and TNF-α [*t*(8) = 1.464, *p* = 0.0907] were similar in early ME7 prion mice (12-wpi) challenged with *S. typhimurium* compared to saline injection (Figures 3A,B). Analysis of mice infected at mid-stage disease, showed significant increases in expression of cytokines following systemic infection with *S. typhimurium* [IL-1β [*t*(7) = 2.090, *p* = 0.0375] and TNF-α [*t*(7) = 2.126, *p* = 0.0355] (Figures 3C,D). This increase in proinflammatory cytokine production following infection with *S. typhimurium* suggests exaggerated activation of microglia because of systemic bacterial infection. These changes in neuroinflammation are only observed when mice are infected at the mid-stage of disease, when loss of synapses and underlying neuropathology is already present.

### Changes in Synaptic Gene Expression Are Dependent on ME7 Prion Disease Progression

**FIGURE 4:**
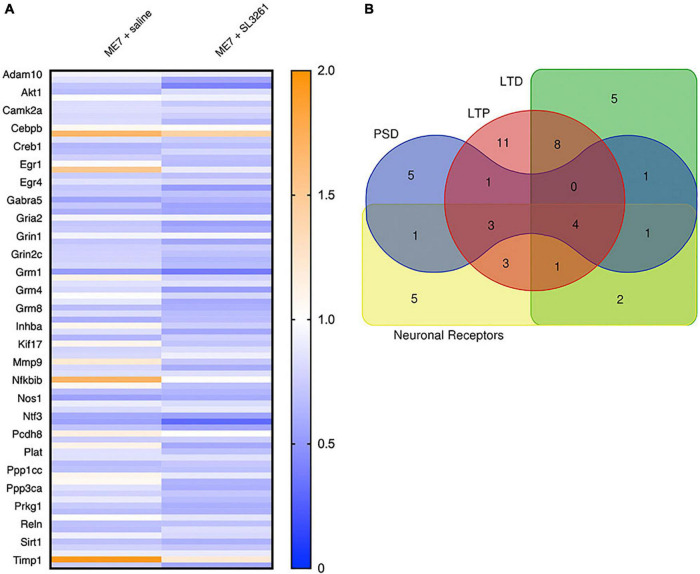
Heatmap and gene associations. Analysis of synaptic plasticity genes in ME7 prion mice at 12-wpi following infection with *S. typhimurium SL3261*. **(A)** Expression of 84 genes with functions linked to synaptic plasticity, including genes associated with long-term potentiation (LTP), long-term depression (LTD), the post-synaptic density (PSD) and neuronal receptors. **(B)** Overlap of genes associated with specific functions in synaptic plasticity.

In the ME7 mouse model, microglial activation is observed from 8 weeks (Betmouni et al., 1996), while the first changes to neuronal networks, including synaptic loss, are detected from 12-wpi (Gray et al., 2009; Hilton et al., 2013). To investigate the effect of a systemic bacterial challenge on synapses in the hippocampus and thalamus, we employed use of a PCR profile array specifically targeted at changes in genes associated with synaptic plasticity. Results were compared to expression with mice injected with normal brain homogenate (NBH) (Figure 4). This array was composed of 84 genes central to synaptic plasticity, including genes associated with structure of the post-synaptic density and those involved in long-term potentiation (LTP) and long-term depression (LTD). Most genes associated with synaptic plasticity show downregulation in ME7 prion compared to NBH-injected mice (Figure 4A and Supplementary Table 2). We identified 5 genes (*Cebpd, Egr2, Mmp9, Nfkbib, Timp1*) that are significantly upregulated in ME7 prion compared to NBH-injected mice (Figure 4A and Supplementary Table 2). These genes are associated with early response in synaptic plasticity and extracellular matrix structure, and the expression levels were not further modified by systemic infection. Genes associated with the post-synaptic density, including *Arc, Dlg4* and *Synpo*, showed decreased expression in ME7 prion mice, but no further decrease following systemic infection (Supplementary Table 2). Analysis of activity-related neuronal receptors which are involved in development of LTP and LTD, such as *Grm1* and *Grin2a*, showed a decrease in response to ME7 prion disease (Supplementary Table 2) and were significantly lower following systemic exposure to *S. typhimurium*.

**FIGURE 5:**
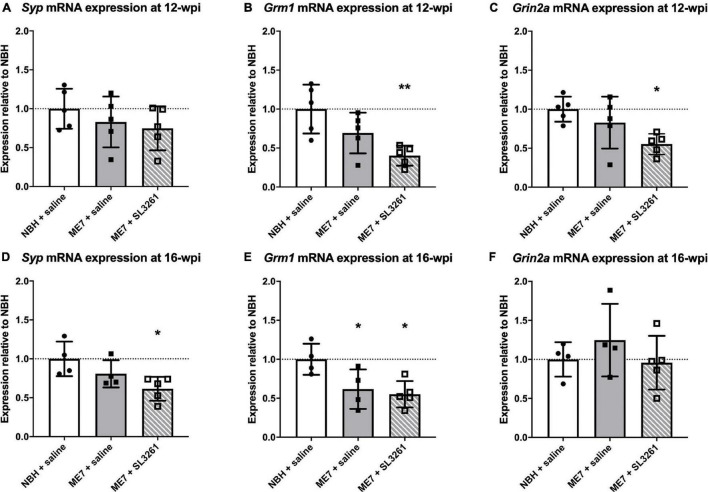
Synapse marker gene transcription. The impact of systemic bacterial infection on synapse-associated gene transcription in ME7 prion mice at 12-wpi **(A–C)** and at 16-wpi **(D–F)** 4 weeks after treatment injection with saline or *S. typhimurium* SL3261 (1 × 10^6^ cfu, i.p.) compared to NBH-injected control mice. **(A)** *Syp* mRNA expression is not reduced in ME7 prion mice at 12-wpi compared to NBH-injected control mice. *Grm1* **(B)** and *Grin2a* **(C)** mRNA expression is significantly reduced following infection in ME7 prion mice compared to NBH-injected control mice. *Syp* **(D)** and *Grm1* **(E)** mRNA expression is significantly reduced at in ME7 prion mice at 16-wpi following infection compared to NBH-injected control mice. *Grin2a* **(F)** mRNA expression at 16-wpi remains stable in ME7 prion mice, even after infection, compared to NBH-injected control mice. *p < 0.05; **p < 0.01 vs. NBH + saline using one-way ANOVA with Holm-Sidak test for multiple comparisons; *n* = 4–5/group.

To confirm and extend the analysis of genes associated with activity-related neuronal receptors, we measured mRNA levels of *Grm1* and *Grin2a* in ME7 prion mice infected with *S. typhimurium* at 12-wpi or 16-wpi. Analysis of mRNA transcript levels confirm a decrease in expression at early stage of disease (12-wpi) for *Grm1* [*F*(2, 12) = 7.306; *p* = 0.0084] and *Grin2a* [*F*(2, 12) = 4.964; *p* = 0.0269], with *post hoc* analysis highlighting significantly lower expression 4 weeks after *S. typhimurium* infection compared to NBH control mice (Figures 5B,C). *Grin2a* expression was not modified when mice were infected at mid-stage disease (16-wpi) [*F*(2, 10) = 0.8174, *p* = 0.4690], whilst *Grm1* expression is significantly reduced after infection at both early and midstage of disease [*F*(2, 10) = 5.843, *p* = 0.0209] (Figures 5E,F). To confirm changes to neurons, we analyzed expression of a pre-synaptic marker, synaptophysin (*Syp*; Figures 5A,D), as neuropathology in ME7 prion mice is known to preferentially affect the pre-synapse (Gray et al., 2009; Siskova et al., 2009; Hilton et al., 2013). Synaptophysin expression is reduced following systemic challenge with *S. typhimurium* at mid stage disease (16-wpi) [*F*(2, 10) = 4.929, *p* = 0.0324], but no changes were observed following infection at early stage disease (12-wpi) [*F*(2, 12) = 0.9821, *p* = 0.4027].

**FIGURE 6:**
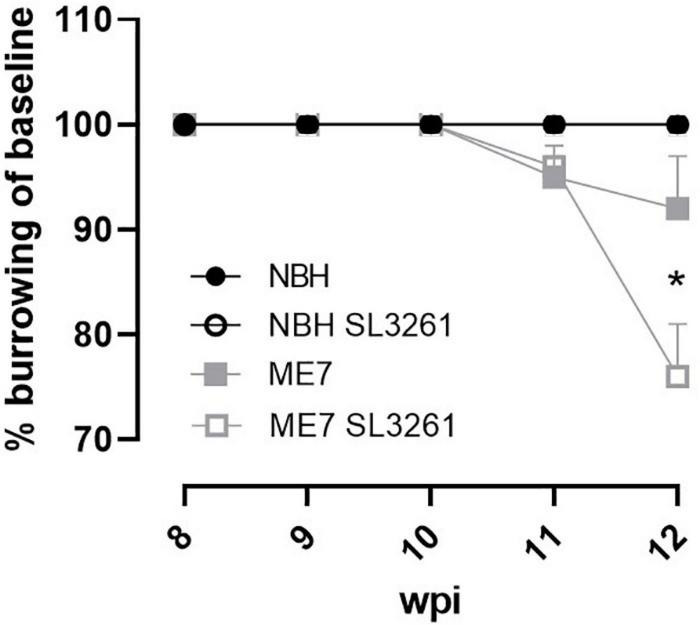
Burrowing activity. The impact of systemic bacterial infection on burrowing behaviors in ME7 prion mice. ME7 prion mice were challenged with saline (closed squares) or 1 × 10^6^ colony forming units *S. typhimurium* SL3261 (open squares) at 8 weeks after inoculation and followed for 4 weeks. NBH-injected mice were included as control. *p < 0.05 using one-way ANOVA with Holm-Sidak test for multiple comparisons; *n* = 4–5/group.

Finally, to investigate if changes in activity-related neuronal receptors are linked to altered behavior, we measured burrowing activity, a behavior that is decreased from 8 weeks in ME7 prion disease (Chouhan et al., 2017). Burrowing was measured on a weekly basis and showed a decrease as the disease progressed (Figure 6). ME7 prion mice, exposed to systemic bacterial infection from 8-wpi significantly alter the rate of burrowing deficit compared to saline-treated mice, measured 4 weeks later (*p* < 0.05). Mice inoculated with normal brain homogenate (NBH) did not show any changes to burrowing activity when exposed to saline or *S. typhimurium*.

## Discussion

Previous studies investigating the response of ME7 prion mice to systemic inflammation have used the bacterial mimetic, LPS. These studies have reported acute changes to cytokine expression in disease-affected brain regions and exaggerated behavioral changes and neuronal loss (Combrinck et al., 2002; Cunningham et al., 2005b; Lunnon et al., 2011; Murray et al., 2011, 2012; Griffin et al., 2013). The aim of this work was to understand the effects of a real, live systemic bacterial infection in the ME7 prion model. We show that systemic infection with the attenuated bacterial strain of *S. typhimurium* (SL3261) results in enhanced CD11b and MHCII expression and cytokine production in the brain at the early stages of disease, which are associated with altered mRNA expression of selected synaptic markers.

### Changes to Microglia in ME7 Prion Mice Following Systemic Infection

We demonstrate that ME7 prion mice show increased neuroinflammation and reduced expression of selected synaptic markers, following systemic infection with *S. typhimurium* when compared to ME7 prion mice treated with saline. These observations confirm that a low-grade systemic bacterial infection can exaggerate neuropathology in the ME7 prion model of neurodegeneration, however, the extent and nature of the changes are dependent on the stage of disease. These results are in accordance with our previous study where we show that systemic infection of naïve mice with *S. typhimurium* results in a prolonged cerebrovascular and neuroinflammatory response (Puntener et al., 2012). Neuropathology in ME7 prion model is characterized by early deposition of PrP^Sc^, microgliosis, loss of synaptophysin density within the molecular layers of the hippocampus and subsequent neuronal loss (Cunningham et al., 2003; Hilton et al., 2013). Increased expression of microglial activation markers and related morphological changes can be detected in ME7 mice from 8-wpi by assessing CD11b expression levels (Betmouni et al., 1996; Gomez-Nicola et al., 2013). Here we show that systemic infection with *S. typhimurium* results in a 1.6-fold increase when mice are infected at 8 wpi and analyzed 4 weeks later (Figure 1). A greater increase in CD11b expression was observed as disease progresses, with a 3.1-fold increase in expression over saline-treated ME7 prion mice, when mice are exposed to infected at 12-wpi and analyzed at 16-wpi (Figure 2). Systemic infection at the early stage of disease, induced a sixfold higher expression of MHCII, while infection at mid stage disease resulted in a twofold increase in expression. This difference in expression may be explained by the relative high expression of MHCII associated with the vasculature. Systemic infection with *S. typhimurium* results in the activation of endothelial cells which contributes to the crosstalk between the peripheral and central immune responses, with increased expression of cytokines and chemokines (van Sorge et al., 2011). Therefore, in addition to the effect on microglia, the activation of the cerebral vasculature may contribute to the enhanced neuropathology and neuroinflammation following infection with *S. typhimurium*. Vascular activation and cerebrovascular dysfunction may precede onset of neurodegeneration (Bell and Zlokovic, 2009; Sweeney et al., 2018), but if observations in the ME7 prion model translate to other neurodegenerative diseases requires further study. Our data suggest that systemic infection may enhance cerebrovascular dysfunction and neuroinflammation at the early stage of ME7 prion disease. Further functional studies, for example disruption of the blood brain barrier and/or infiltration of immune cells are required to identify underlying mechanisms. We cannot exclude that enhanced neuropathology is due to altered prion levels in the brain, and future studies will be required to investigate if microbial infections influence prion replication or seeding activity.

### Functional Changes to Microglia in ME7 Prion Mice Following Systemic Infection

Previous studies have shown that microglia are primed by ME7 prion-driven neuropathology and respond with a greater potency to inflammatory stimuli compared to naïve microglia (Cunningham et al., 2009; Murray et al., 2012; Griffin et al., 2013; Skelly et al., 2019). Activation of primed microglia with central or systemic LPS results in exaggerated and prolonged cellular activation and production of the pro-inflammatory cytokines IL-1β and TNF-α (Cunningham et al., 2005b,^2009^). In this study we investigated if a real, live bacterial infection induces similar functional changes to microglia. Infection at early stage did not induce a significant change in cytokines production and IL-1β and TNF-α expression levels measured in hippocampal/thalamic were comparable to ME7 mice treated with saline. However, systemic infection of ME7 prion mice at mid-stage resulted in a significant increase in both IL-1β and TNF-α mRNA transcripts measured 4 weeks later (Figure 3). Microglial priming can occur because of misfolded protein deposition and/or the loss of synaptic density in the hippocampus. The latter is a neuropathological hallmark which has not yet taken place at 8-wpi. Microglia in the healthy brain contribute to homeostasis more effectively than primed microglia (Lim et al., 2015; Neher and Cunningham, 2019). Thus, it is possible that ME7 prion mice that were infected at early stage show a temporal profile of cytokine production similar to a naïve mouse. In naïve mice central cytokine expression peaks at 21 days following systemic bacterial challenge (Puntener et al., 2012), and measurement after 4 weeks may not capture this response. Microglial numbers in the hippocampus and thalamus increase in ME7 prion mice from 12-wpi (Gomez-Nicola et al., 2013). The differences in cytokine production in ME7 prion mice challenged at early and mid-stage disease could therefore also be explained by an increase in microglia numbers. Together, these data suggest that the exaggerated effects of systemic inflammation on cytokine production in the ME7 prion model are only present at the mid-stage of disease when higher number of microglia are present, along with concurrent protein deposition and synaptic pathology (Cunningham et al., 2005a,^2009^; Gray et al., 2009; Hilton et al., 2013).

### Changes to Synaptic Plasticity in ME7 Prion Mice Following Systemic Infection

Synaptic density in the *stratum radiatum* of the hippocampal CA1 is reduced in ME7 prion mice from 12 to 13 wpi, coinciding with behavioral deficits and motor function (Jeffrey et al., 2000; Hilton et al., 2013). Synaptic dysfunction and/or loss in the ME7 prion model has been described predominantly in the pre-synaptic compartment, mostly by measuring synaptophysin expression levels, although alterations to the post-synaptic density are also observed (Gray et al., 2009; Siskova et al., 2009, 2010). To investigate the effect of a bacterial infection on synaptic markers we analyzed the gene expression of a range of molecules linked to synaptic plasticity (Supplementary Table 2). We found reduced expression of gene transcription related to synaptic plasticity in ME7 prion mice, including LTP, LTD, postsynaptic density, neuronal receptors (in particular glutamate receptors) and other structural and plasticity genes (Table 2 and additionally Supplementary Table 2). Importantly, we found further reduction of these selected molecules in ME7 mice exposed to a bacterial infection. Loss of the perineuronal net is evident in ME7 prion mice at 16-wpi (Franklin et al., 2008; McRae and Porter, 2012). Given that profile array data shows a decrease in *Ncam1*, GABAR α5 subunit and AMPAR subunit expression, along with increased *Mmp9* expression (Supplementary Table 2), it could be postulated that these are all indicative of early perineuronal net breakdown in ME7 prion mice after systemic bacterial challenge at 8-wpi. Loss of the perineuronal net may therefore detrimentally affect extra-synapse glutamate receptor expression (e.g., *Grm1*) as opposed to post-synaptic receptors (e.g., *Grin2a*). Together, these data do suggest that synaptic expression of glutamate receptors is altered in ME7 prion mice and further reduced following exposure to systemic bacterial infection.

**TABLE 2:** Functional gene group changes.

Pathway description (number of genes)	ME7 + saline	ME7 + SL3261
Long-term potentiation (*n* = 31)	0.7992	0.6790
Long-term depression (*n* = 22)	0.7845	0.6899
Postsynaptic density (*n* = 16)	0.7913	0.7188
Neuronal receptors (*n* = 20)	0.7823	0.6623
Other structural/plasticity genes (*n* = 32)	0.9506	0.8011

*Fold change in expression compared to NBH mice 4 weeks after systemic bacterial challenge with S. typhimurium SL3261 at 8-wpi in ME7 prion mice.*

To further investigate if these changes to synaptic markers depend on the time during disease progression, we measured expression of selected genes in mice infected at different stages of disease (Figure 5). We found an expected decrease in *Syp* expression levels in ME7 mice at 12 wpi, which was further decreased when mice were exposed to a systemic bacterial infection. No changes were observed in mice infected at early stage, suggesting that the bacterial infection *per se* does not impact Syp expression, but in the presence of an ongoing neurodegenerative disease it enhances synaptic degeneration. Previous studies showed no change in expression of glutamate receptors (NMDARs) in ME7 prion mice, when compared to control mice (Gray et al., 2009). We confirmed these observations by analyzing mRNA transcript levels for the N2a NMDAR subunit (*Grin2a*). We further show that systemic bacterial infection results in a significant reduction of *Grin2a* expression, especially at the early stage of ME7 prion disease. This response is associated with reduced levels of mGluR1 (*Grm1*). Both mGluR1 and the N2a NMDR are expressed at the post-synaptic compartment of neurons and the induction of persistent long-term potentiation (LTP) is dependent on activation of these receptors (van Dam et al., 2004; Ferraguti and Shigemoto, 2006; Cheyne and Montgomery, 2008; Mukherjee and Manahan-Vaughan, 2013). Our data suggest that systemic bacterial infection exaggerates the loss of glutamate receptor expression, which may explain reduced neuronal function and earlier onset and/or progression of neuropathology as observed with changes in burrowing activity. Interestingly, these changes to behavior and glutamate receptors occurred in the absence of enhanced IL-1β and TNF-α production.

A number of previous studies used the bacterial mimetic LPS to address the effect of systemic inflammation on AD-like neuropathology. These experiments have yielded conflicting results that seem to depend on dose, timing, and frequency of LPS administration (Wendeln et al., 2018; Tejera et al., 2019). The effect of a live systemic bacterial infection or bacterial sepsis has also been studied, using experimental models of neurodegeneration. For example, oral application of live *Porphyromonas gingivalis* significantly enhances amyloid and tau pathology, neuroinflammation and cognitive impairment in aged wild type mice and APP/PS1 transgenic mice (Ding et al., 2018; Ilievski et al., 2018; Dominy et al., 2019) and a real live *Bordetella pertussis* respiratory infection results in increased neuroinflammation and Aβ_40_ load (McManus et al., 2014). Interestingly, recurrent, acute systemic infections with *Streptococcus pneumoniae* do not alter the onset or course of the disease when tested in various experimental models of neurodegeneration (Ebert et al., 2010), suggesting that enhanced neurodegeneration may only occur following sustained, chronic infections. The impact of polymicrobial sepsis on the brain has also been investigated. For example, cecal ligation results in sustained neuroinflammation in wild type mice (Singer et al., 2016) and amyloid plaque deposition in APP/PS1-21 transgenic mice (Basak et al., 2021). Cognitive deficits were also observed, but these occurred in the absence of neuronal loss or changes in synaptic density in the hippocampus (Singer et al., 2016). In this study we show that a real live, low-grade *Salmonella typhimurium* infection enhances neuroinflammation and synaptic loss in the ME7 prion model and that pre-existing neuronal damage may be needed for bacteria to enhance deterioration. Our study used adult mice and did not investigate the effect of aging. Bento-Torres et al. (2017) showed that older animals show a more robust immune response to prion disease, but if older animals with ME7 prion disease show further enhanced neuropathology remains to be investigated.

## Summary

In summary, we confirm that ME7 prion disease results in a robust neuroinflammation and shows an exaggerated innate immune response in the brain following systemic exposure to a real, live bacterial infection. Morphological and functional changes to microglia were most evident as the disease progressed. This exaggerated response could be due to increased number of microglia, their activation state (i.e., primed) or the level of neuropathology at the time of infection. Systemic bacterial challenge in ME7 prion mice with *S. typhimurium* at early stage did not result in enhanced cytokine production, likely due to low level of pathology and microglial activation or numbers at this timepoint. Systemic bacterial infection also modified gene expression of various biological pathways involved in synaptic plasticity, and in particular glutamate receptors. These changes were already observed at the early stage of disease. Our results provide new insight into the effect of a systemic bacterial infection during neurodegeneration and provide an explanation why common bacterial infections, such as urinary tract infection or gum disease are risk factors for earlier onset and/or progression of cognitive decline in people with dementia.

## Data Availability Statement

The original contributions presented in the study are included in the article/Supplementary Material, further inquiries can be directed to the corresponding author/s.

## Ethics Statement

All procedures were reviewed and approved from the University of Southampton UK Animal Welfare and Ethical Review Body (AWERB) and were performed in accordance with Home Office project licenses (30/3056, 30/3057) under the United Kingdom Animals (Scientific Procedures) Act (1986).

## Author Contributions

JC performed analysis of cytokines and synaptic markers, analyzed, and quantified histology data and drafted the manuscript. UP and SB performed the animal and histological experiments and analyzed the data. UP and JT contributed to study design. JT edited the manuscript, supervised the work, and received grant funding. All authors contributed to the article and approved the submitted version.

## Conflict of Interest

The handling editor declared a past co-authorship with one of the authors JT. The authors declare that the research was conducted in the absence of any commercial or financial relationships that could be construed as a potential conflict of interest.

## Publisher’s Note

All claims expressed in this article are solely those of the authors and do not necessarily represent those of their affiliated organizations, or those of the publisher, the editors and the reviewers. Any product that may be evaluated in this article, or claim that may be made by its manufacturer, is not guaranteed or endorsed by the publisher.

## Supplementary Material

Supplementary Figure 1 Body weight changes following systemic infection with *S. typhimurium.* ME7 prion mice received an i.p injection of with saline (closed squares) or 1 × 10^6^ colony forming units *S. typhimurium* SL3261 (open squares) at 8 weeks after ME7 inoculation and body weight of mice was monitored for 4 weeks. NBH-injected mice (closed circles) were included as control. ^∗∗∗^*p* < 0.001 between ME7 + SL3261 and NBH mice at 1-day post-infection; *n* = 4–5/group plotted as mean ± SEM.

## References

[B1] Anand R. Gill K. D. Mahdi A. A. (2014). Therapeutics of Alzheimer’s disease: past, present and future. *Neuropharmacology* 76 27–50. 10.1016/j.neuropharm.2013.07.004 23891641

[B2] Basak J. M. Ferreiro A. Cohen L. S. Sheehan P. W. Nadarajah C. J. Kanan M. F. (2021). Bacterial sepsis increases hippocampal fibrillar amyloid plaque load and neuroinflammation in a mouse model of Alzheimer’s disease. *Neurobiol. Dis.* 152:105292. 10.1016/j.nbd.2021.105292 33556539 PMC8057119

[B3] Bell R. D. Zlokovic B. V. (2009). Neurovascular mechanisms and blood-brain barrier disorder in Alzheimer’s disease. *Acta Neuropathol.* 118 103–113. 10.1007/s00401-009-0522-3 19319544 PMC2853006

[B4] Bento-Torres J. Sobral L. L. Reis R. R. de Oliveira R. B. Anthony D. C. Vasconcelos P. F. (2017). Age and Environment Influences on Mouse Prion Disease Progression: behavioral Changes and Morphometry and Stereology of Hippocampal Astrocytes. *Oxid. Med. Cell. Longev.* 2017:4504925. 10.1155/2017/4504925 28243355 PMC5294381

[B5] Betmouni S. Perry V. H. Gordon J. L. (1996). Evidence for an early inflammatory response in the central nervous system of mice with scrapie. *Neuroscience* 74 1–5. 10.1016/0306-4522(96)00212-6 8843071

[B6] Butchart J. Brook L. Hopkins V. Teeling J. Puntener U. Culliford D. (2015). Etanercept in Alzheimer disease: a randomized, placebo-controlled, double-blind, phase 2 trial. *Neurology* 84 2161–2168. 10.1212/WNL.0000000000001617 25934853 PMC4451045

[B7] Cheyne J. E. Montgomery J. M. (2008). Plasticity-dependent changes in metabotropic glutamate receptor expression at excitatory hippocampal synapses. *Mol. Cell. Neurosci.* 37 432–439. 10.1016/j.mcn.2007.10.015 18191411

[B8] Chiti Z. Knutsen O. M. Betmouni S. Greene J. R. (2006). An integrated, temporal study of the behavioural, electrophysiological and neuropathological consequences of murine prion disease. *Neurobiol. Dis.* 22 363–373. 10.1016/j.nbd.2005.12.002 16431123

[B9] Chouhan J. K. Fowler S. B. Webster C. I. Teeling J. L. (2017). The ME7 prion model of neurodegeneration as a tool to understand and target neuroinflammation in Alzheimer’s disease. *Drug Discov. Today Dis. Models* 2 45–52. 10.1016/j.ddmod.2018.10.004

[B10] Combrinck M. I. Perry V. H. Cunningham C. (2002). Peripheral infection evokes exaggerated sickness behaviour in pre-clinical murine prion disease. *Neuroscience* 112 7–11. 10.1016/s0306-4522(02)00030-1 12044467

[B11] Cunningham C. Campion S. Lunnon K. Murray C. L. Woods J. F. Deacon R. M. (2009). Systemic inflammation induces acute behavioral and cognitive changes and accelerates neurodegenerative disease. *Biol. Psychiatry* 65 304–312. 10.1016/j.biopsych.2008.07.024 18801476 PMC2633437

[B12] Cunningham C. Deacon R. Wells H. Boche D. Waters S. Diniz C. P. (2003). Synaptic changes characterize early behavioural signs in the ME7 model of murine prion disease. *Eur. J. Neurosci.* 17 2147–2155. 10.1046/j.1460-9568.2003.02662.x 12786981

[B13] Cunningham C. Wilcockson D. C. Campion S. Lunnon K. Perry V. H. (2005b). Central and systemic endotoxin challenges exacerbate the local inflammatory response and increase neuronal death during chronic neurodegeneration. *J. Neurosci.* 25 9275–9284. 10.1523/JNEUROSCI.2614-05.2005 16207887 PMC6725757

[B14] Cunningham C. Deacon R. M. Chan K. Boche D. Rawlins J. N. Perry V. H. (2005a). Neuropathologically distinct prion strains give rise to similar temporal profiles of behavioral deficits. *Neurobiol. Dis.* 18 258–269. 10.1016/j.nbd.2004.08.015 15686954

[B15] Ding Y. Ren J. Yu H. Yu W. Zhou Y. (2018). Porphyromonas gingivalis, a periodontitis causing bacterium, induces memory impairment and age-dependent neuroinflammation in mice. *Immun. Ageing* 15:6. 10.1186/s12979-017-0110-7 29422938 PMC5791180

[B16] Dominy S. S. Lynch C. Ermini F. Benedyk M. Marczyk A. Konradi A. (2019). Porphyromonas gingivalis in Alzheimer’s disease brains: evidence for disease causation and treatment with small-molecule inhibitors. *Sci. Adv.* 5:eaau3333. 10.1126/sciadv.aau3333 30746447 PMC6357742

[B17] Dursun E. Gezen-Ak D. Hanagasi H. Bilgic B. Lohmann E. Ertan S. (2015). The interleukin 1 alpha, interleukin 1 beta, interleukin 6 and alpha-2-macroglobulin serum levels in patients with early or late onset Alzheimer’s disease, mild cognitive impairment or Parkinson’s disease. *J. Neuroimmunol.* 283 50–57. 10.1016/j.jneuroim.2015.04.014 26004156

[B18] Ebert S. Goos M. Rollwagen L. Baake D. Zech W. D. Esselmann H. (2010). Recurrent systemic infections with Streptococcus pneumoniae do not aggravate the course of experimental neurodegenerative diseases. *J. Neurosci. Res.* 88 1124–1136. 10.1002/jnr.22270 19859962

[B19] Ferraguti F. Shigemoto R. (2006). Metabotropic glutamate receptors. *Cell Tissue Res.* 326 483–504. 10.1007/s00441-006-0266-5 16847639

[B20] Field R. Campion S. Warren C. Murray C. Cunningham C. (2010). Systemic challenge with the TLR3 agonist poly I:C induces amplified IFNalpha/beta and IL-1beta responses in the diseased brain and exacerbates chronic neurodegeneration. *Brain Behav. Immun.* 24 996–1007. 10.1016/j.bbi.2010.04.004 20399848 PMC3334265

[B21] Franklin S. L. Love S. Greene J. R. Betmouni S. (2008). Loss of Perineuronal Net in ME7 Prion Disease. *J. Neuropathol. Exp. Neurol.* 67 189–199. 10.1097/NEN.0b013e3181654386 18344910

[B22] Gomez-Nicola D. Fransen N. L. Suzzi S. Perry V. H. (2013). Regulation of microglial proliferation during chronic neurodegeneration. *J. Neurosci.* 33 2481–2493. 10.1523/JNEUROSCI.4440-12.2013 23392676 PMC6619184

[B23] Gray B. C. Siskova Z. Perry V. H. O’Connor V. (2009). Selective presynaptic degeneration in the synaptopathy associated with ME7-induced hippocampal pathology. *Neurobiol. Dis.* 35 63–74. 10.1016/j.nbd.2009.04.001 19362593

[B24] Griffin E. W. Skelly D. T. Murray C. L. Cunningham C. (2013). Cyclooxygenase-1-dependent prostaglandins mediate susceptibility to systemic inflammation-induced acute cognitive dysfunction. *J. Neurosci.* 33 15248–15258. 10.1523/JNEUROSCI.6361-11.2013 24048854 PMC3776067

[B25] Heneka M. T. Carson M. J. El Khoury J. Landreth G. E. Brosseron F. Feinstein D. L. (2015). Neuroinflammation in Alzheimer’s disease. *Lancet Neurol.* 14 388–405. 10.1016/S1474-4422(15)70016-5 25792098 PMC5909703

[B26] Hennessy E. Griffin E. W. Cunningham C. (2015). Astrocytes Are Primed by Chronic Neurodegeneration to Produce Exaggerated Chemokine and Cell Infiltration Responses to Acute Stimulation with the Cytokines IL-1beta and TNF-alpha. *J. Neurosci.* 35 8411–8422. 10.1523/JNEUROSCI.2745-14.2015 26041910 PMC4452550

[B27] Hilton K. J. Cunningham C. Reynolds R. A. Perry V. H. (2013). Early Hippocampal Synaptic Loss Precedes Neuronal Loss and Associates with Early Behavioural Deficits in Three Distinct Strains of Prion Disease. *PLoS One* 8:e68062. 10.1371/journal.pone.0068062 23840812 PMC3694005

[B28] Holmes C. Cunningham C. Zotova E. Culliford D. Perry V. H. (2011). Proinflammatory cytokines, sickness behavior, and Alzheimer disease. *Neurology* 77 212–218. 10.1212/WNL.0b013e318225ae07 21753171 PMC3136056

[B29] Holmes C. Cunningham C. Zotova E. Woolford J. Dean C. Kerr S. (2009). Systemic inflammation and disease progression in Alzheimer disease. *Neurology* 73 768–774. 10.1212/WNL.0b013e3181b6bb95 19738171 PMC2848584

[B30] Holmes C. El-Okl M. Williams A. L. Cunningham C. Wilcockson D. Perry V. H. (2003). Systemic infection, interleukin 1beta, and cognitive decline in Alzheimer’s disease. *J. Neurol. Neurosurg. Psychiatry* 74 788–789. 10.1136/jnnp.74.6.788 12754353 PMC1738504

[B31] Ibbett P. Goverdhan S. V. Pipi E. Chouhan J. K. Keeling E. Angus E. M. (2019). A lasered mouse model of retinal degeneration displays progressive outer retinal pathology providing insights into early geographic atrophy. *Sci. Rep.* 9:7475. 10.1038/s41598-019-43906-z 31097765 PMC6522499

[B32] Ide M. Harris M. Stevens A. Sussams R. Hopkins V. Culliford D. (2016). Periodontitis and Cognitive Decline in Alzheimer’s Disease. *PLoS One* 11:e0151081. 10.1371/journal.pone.0151081 26963387 PMC4786266

[B33] Ilievski V. Zuchowska P. K. Green S. J. Toth P. T. Ragozzino M. E. Le K. (2018). Chronic oral application of a periodontal pathogen results in brain inflammation, neurodegeneration and amyloid beta production in wild type mice. *PLoS One* 13:e0204941. 10.1371/journal.pone.0204941 30281647 PMC6169940

[B34] Jeffrey M. Halliday W. G. Bell J. Johnston A. R. MacLeod N. K. Ingham C. (2000). Synapse loss associated with abnormal PrP precedes neuronal degeneration in the scrapie-infected murine hippocampus. *Neuropathol. Appl. Neurobiol.* 26 41–54. 10.1046/j.1365-2990.2000.00216.x 10736066

[B35] Lim S. L. Rodriguez-Ortiz C. J. Kitazawa M. (2015). Infection, systemic inflammation, and Alzheimer’s disease. *Microbes Infect.* 17 549–556. 10.1016/j.micinf.2015.04.004 25912134

[B36] Lunnon K. Teeling J. L. Tutt A. L. Cragg M. S. Glennie M. J. Perry V. H. (2011). Systemic inflammation modulates Fc receptor expression on microglia during chronic neurodegeneration. *J. Immunol.* 186 7215–7224. 10.4049/jimmunol.0903833 21572034

[B37] McManus R. M. Higgins S. C. Mills K. H. Lynch M. A. (2014). Respiratory infection promotes T cell infiltration and amyloid-beta deposition in APP/PS1 mice. *Neurobiol. Aging* 35 109–121. 10.1016/j.neurobiolaging.2013.07.025 23993702

[B38] McRae P. A. Porter B. E. (2012). The perineuronal net component of the extracellular matrix in plasticity and epilepsy. *Neurochem. Int.* 61 963–972. 10.1016/j.neuint.2012.08.007 22954428 PMC3930202

[B39] Mukherjee S. Manahan-Vaughan D. (2013). Role of metabotropic glutamate receptors in persistent forms of hippocampal plasticity and learning. *Neuropharmacology* 66 65–81. 10.1016/j.neuropharm.2012.06.005 22743159

[B40] Murray C. Sanderson D. J. Barkus C. Deacon R. M. Rawlins J. N. Bannerman D. M. (2012). Systemic inflammation induces acute working memory deficits in the primed brain: relevance for delirium. *Neurobiol. Aging* 33 603–616.e3. 10.1016/j.neurobiolaging.2010.04.002 20471138 PMC3200140

[B41] Murray C. L. Skelly D. T. Cunningham C. (2011). Exacerbation of CNS inflammation and neurodegeneration by systemic LPS treatment is independent of circulating IL-1beta and IL-6. *J. Neuroinflammation* 8:50. 10.1186/1742-2094-8-50 21586125 PMC3119173

[B42] Neher J. J. Cunningham C. (2019). Priming Microglia for Innate Immune Memory in the Brain. *Trends Immunol.* 40 358–374. 10.1016/j.it.2019.02.001 30833177

[B43] Ott B. R. Jones R. N. Daiello L. A. de la Monte S. M. Stopa E. G. Johanson C. E. (2018). Blood-Cerebrospinal Fluid Barrier Gradients in Mild Cognitive Impairment and Alzheimer’s Disease: relationship to Inflammatory Cytokines and Chemokines. *Front. Aging Neurosci.* 10:245. 10.3389/fnagi.2018.00245 30186149 PMC6110816

[B44] Peters S. E. Paterson G. K. Bandularatne E. S. Northen H. C. Pleasance S. Willers C. (2010). *Salmonella enterica* serovar typhimurium trxA mutants are protective against virulent challenge and induce less inflammation than the live-attenuated vaccine strain SL3261. *Infect. Immun.* 78 326–336. 10.1128/IAI.00768-09 19884329 PMC2798184

[B45] Puntener U. Booth S. G. Perry V. H. Teeling J. L. (2012). Long-term impact of systemic bacterial infection on the cerebral vasculature and microglia. *J. Neuroinflammation* 9:146. 10.1186/1742-2094-9-146 22738332 PMC3439352

[B46] Rakic S. Hung Y. M. A. Smith M. So D. Tayler H. M. Varney W. (2018). Systemic infection modifies the neuroinflammatory response in late stage Alzheimer’s disease. *Acta Neuropathol. Commun.* 6:88. 10.1186/s40478-018-0592-3 30193587 PMC6127939

[B47] Rueden C. T. Schindelin J. Hiner M. C. DeZonia B. E. Walter A. E. Arena E. T. (2017). ImageJ2: imageJ for the next generation of scientific image data. *BMC Bioinformatics* 18:529. 10.1186/s12859-017-1934-z 29187165 PMC5708080

[B48] Ruifrok A. C. Johnston D. A. (2001). Quantification of histochemical staining by color deconvolution. *Anal. Quant. Cytol. Histol.* 23 291–299. 11531144

[B49] Singer B. H. Newstead M. W. Zeng X. Cooke C. L. Thompson R. C. Singer K. (2016). Cecal Ligation and Puncture Results in Long-Term Central Nervous System Myeloid Inflammation. *PLoS One* 11:e0149136. 10.1371/journal.pone.0149136 26862765 PMC4749127

[B50] Siskova Z. Page A. O’Connor V. Perry V. H. (2009). Degenerating synaptic boutons in prion disease: microglia activation without synaptic stripping. *Am. J. Pathol.* 175 1610–1621. 10.2353/ajpath.2009.090372 19779137 PMC2751557

[B51] Siskova Z. Sanyal N. K. Orban A. O’Connor V. Perry V. H. (2010). Reactive hypertrophy of synaptic varicosities within the hippocampus of prion-infected mice. *Biochem. Soc. Trans.* 38 471–475. 10.1042/BST0380471 20298205

[B52] Skelly D. T. Griffin E. W. Murray C. L. Harney S. O’Boyle C. Hennessy E. (2019). Acute transient cognitive dysfunction and acute brain injury induced by systemic inflammation occur by dissociable IL-1-dependent mechanisms. *Mol. Psychiatry* 24 1533–1548. 10.1038/s41380-018-0075-8 29875474 PMC6510649

[B53] Stoeck K. Schmitz M. Ebert E. Schmidt C. Zerr I. (2014). Immune responses in rapidly progressive dementia: a comparative study of neuroinflammatory markers in Creutzfeldt-Jakob disease, Alzheimer’s disease and multiple sclerosis. *J. Neuroinflammation* 11:170. 10.1186/s12974-014-0170-y 25315814 PMC4207356

[B54] Sweeney M. D. Sagare A. P. Zlokovic B. V. (2018). Blood-brain barrier breakdown in Alzheimer disease and other neurodegenerative disorders. *Nat. Rev. Neurol.* 14 133–150. 10.1038/nrneurol.2017.188 29377008 PMC5829048

[B55] Tejera D. Mercan D. Sanchez-Caro J. M. Hanan M. Greenberg D. Soreq H. (2019). Systemic inflammation impairs microglial Abeta clearance through NLRP3 inflammasome. *EMBO J.* 38:e101064. 10.15252/embj.2018101064 31359456 PMC6717897

[B56] van Dam E. J. Kamal A. Artola A. de Graan P. N. Gispen W. H. Ramakers G. M. (2004). Group I metabotropic glutamate receptors regulate the frequency-response function of hippocampal CA1 synapses for the induction of LTP and LTD. *Eur. J. Neurosci.* 19 112–118. 10.1111/j.1460-9568.2004.03103.x 14750969

[B57] van Sorge N. M. Zialcita P. A. Browne S. H. Quach D. Guiney D. G. Doran K. S. (2011). Penetration and activation of brain endothelium by *Salmonella enterica* serovar Typhimurium. *J. Infect. Dis.* 203 401–405. 10.1093/infdis/jiq048 21186258 PMC3071103

[B58] Wendeln A. C. Degenhardt K. Kaurani L. Gertig M. Ulas T. Jain G. (2018). Innate immune memory in the brain shapes neurological disease hallmarks. *Nature* 556 332–338. 10.1038/s41586-018-0023-4 29643512 PMC6038912

