# Supplementary figures and images for: Systemic Inflammation Accelerates Changes in Microglial and Synaptic Markers in an Experimental Model of Chronic Neurodegeneration
Source: Front Neurosci. 2022 Jan 4;15:760721. doi: 10.3389/fnins.2021.760721 (PMC8764443; doi:10.3389/fnins.2021.760721)

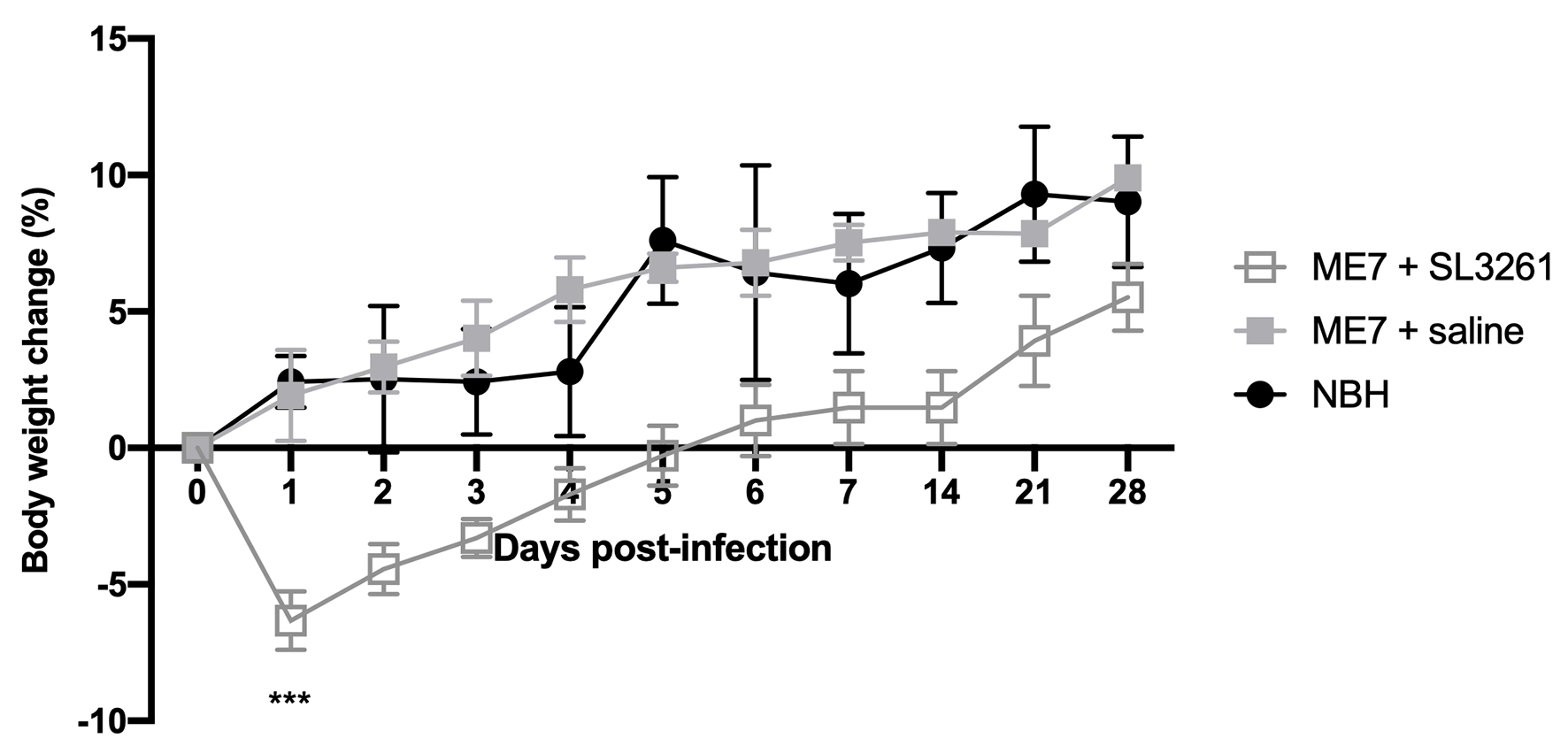

Supplement: Supplementary Figure 1 — Body weight changes following systemic infection with S. typhimurium. ME7 prion mice received an i.p injection of with saline (closed squares) or 1 × 10^6^ colony forming units S. typhimurium SL3261 (open squares) at 8 weeks after ME7 inoculation and body weight of mice was monitored for 4 weeks. NBH-injected mice (closed circles) were included as control. ∗∗∗p < 0.001 between ME7 + SL3261 and NBH mice at 1-day post-infection; n = 4–5/group plotted as mean ± SEM. [file Image_1.TIFF]
