# Supplementary material for: Systemic Inflammation Accelerates Changes in Microglial and Synaptic Markers in an Experimental Model of Chronic Neurodegeneration
Source: Front Neurosci. 2022 Jan 4;15:760721. doi: 10.3389/fnins.2021.760721 (PMC8764443; doi:10.3389/fnins.2021.760721)
Supplement: Supplementary file 2 [file Table_1.DOCX]

**Supplementary Table 1: Specifications for primer design with NCBI-Blast**

|  | Minimum | Optimum | Maximum |
| --- | --- | --- | --- |
| Product length (bp) | 70 |  | 300 |
| Melting temperature (°C) | 57 | 60 | 63 |
| Primer size (bp) | 15 | 20 | 25 |
| Max. self-complementarity | 0 | 0 | 7 |
| Max. self 3’ complementarity | 0 | 0 | 3 |
| Primer GC content (%) | 40 | 50 | 65 |
