# Supplementary material for: Systemic Inflammation Accelerates Changes in Microglial and Synaptic Markers in an Experimental Model of Chronic Neurodegeneration
Source: Front Neurosci. 2022 Jan 4;15:760721. doi: 10.3389/fnins.2021.760721 (PMC8764443; doi:10.3389/fnins.2021.760721)
Supplement: Supplementary file 3 [file Table_2.DOCX]

**Supplementary Table 2: Synaptic Plasticity PCR Profiler**

Fold change in expression compared to NBH mice four weeks after systemic bacterial challenge with *S. typhimurium* SL3261 at 8-wpi in ME7 prion mice.

| Gene | Description | ME7 + saline | ME7 + SL3261 |
| --- | --- | --- | --- |
| Adam10 | A disintegrin and metallopeptidase domain 10 | 0.9369 | 0.8950 |
| Adcy1 | Adenylate cyclase 1 | 0.8562 | 0.5743 |
| Adcy8 | Adenylate cyclase 8 | 0.7150 | 0.4118 |
| Akt1 | Thymoma viral proto-oncogene 1 | 0.6811 | 0.8236 |
| Arc | Activity regulated cytoskeletal-associated protein | 0.9903 | 0.9266 |
| Bdnf | Brain derived neurotrophic factor | 0.8328 | 0.7371 |
| Camk2a | Calcium/calmodulin-dependent protein kinase II alpha | 0.8386 | 0.8236 |
| Camk2g | Calcium/calmodulin-dependent protein kinase II gamma | 0.8156 | 0.7684 |
| Cdh2 | Cadherin 2 | 0.8213 | 0.6598 |
| Cebpb | CCAAT/enhancer binding protein (C/EBP), beta | 1.0614 | 1.0210 |
| Cebpd | CCAAT/enhancer binding protein (C/EBP), delta | 1.6656 | 1.4241 |
| Cnr1 | Cannabinoid receptor 1 (brain) | 0.8503 | 0.7474 |
| Creb1 | CAMP responsive element binding protein 1 | 0.6534 | 0.6830 |
| Crem | CAMP responsive element modulator | 0.6811 | 0.8011 |
| Dlg4 | Discs, large homolog 4 (Drosophila) | 0.7716 | 0.6878 |
| Egr1 | Early growth response 1 | 0.9767 | 0.6690 |
| Egr2 | Early growth response 2 | 1.5220 | 0.8950 |
| Egr3 | Early growth response 3 | 0.7505 | 0.6926 |
| Egr4 | Early growth response 4 | 0.8503 | 0.8123 |
| Ephb2 | Eph receptor B2 | 0.7200 | 0.5249 |
| Fos | FBJ osteosarcoma oncogene | 0.7505 | 0.6926 |
| Gabra5 | Gamma-aminobutyric acid (GABA) A receptor, subunit alpha 5 | 0.5649 | 0.6417 |
| Gnai1 | Guanine nucleotide binding protein (G protein), alpha inhibiting 1 | 0.7250 | 0.5625 |
| Gria1 | Glutamate receptor, ionotropic, AMPA1 (alpha 1) | 0.6096 | 0.5704 |
| Gria2 | Glutamate receptor, ionotropic, AMPA2 (alpha 2) | 0.9500 | 0.9395 |
| Gria3 | Glutamate receptor, ionotropic, AMPA3 (alpha 3) | 0.7200 | 0.5510 |
| Gria4 | Glutamate receptor, ionotropic, AMPA4 (alpha 4) | 0.7454 | 0.6029 |
| Grin1 | Glutamate receptor, ionotropic, NMDA1 (zeta 1) | 0.9369 | 0.9526 |
| Grin2a | Glutamate receptor, ionotropic, NMDA2A (epsilon 1) | 0.7150 | 0.5704 |
| Grin2b | Glutamate receptor, ionotropic, NMDA2B (epsilon 2) | 0.7663 | 0.7474 |
| Grin2c | Glutamate receptor, ionotropic, NMDA2C (epsilon 3) | 0.7770 | 0.6926 |
| Grin2d | Glutamate receptor, ionotropic, NMDA2D (epsilon 4) | 0.7770 | 0.6507 |
| Grip1 | Glutamate receptor interacting protein 1 | 0.8100 | 0.6690 |
| Grm1 | Glutamate receptor, metabotropic 1 | 0.5270 | 0.3816 |
| Grm2 | Glutamate receptor, metabotropic 2 | 1.1142 | 0.7792 |
| Grm3 | Glutamate receptor, metabotropic 3 | 0.8503 | 0.8586 |
| Grm4 | Glutamate receptor, metabotropic 4 | 0.8100 | 0.5359 |
| Grm5 | Glutamate receptor, metabotropic 5 | 0.9903 | 0.8011 |
| Grm7 | Glutamate receptor, metabotropic 7 | 0.8803 | 0.5905 |
| Grm8 | Glutamate receptor, metabotropic 8 | 0.6718 | 0.6113 |
| Homer1 | Homer homolog 1 (Drosophila) | 0.8328 | 0.6690 |
| Igf1 | Insulin-like growth factor 1 | 0.6139 | 0.6783 |
| Inhba | Inhibin beta-A | 1.0837 | 0.7579 |
| Jun | Jun oncogene | 0.8386 | 0.5783 |
| Junb | Jun-B oncogene | 0.6399 | 0.7684 |
| Kif17 | Kinesin family member 17 | 1.0837 | 0.7120 |
| Klf10 | Kruppel-like factor 10 | 0.7879 | 0.8293 |
| Mapk1 | Mitogen-activated protein kinase 1 | 0.8100 | 0.9202 |
| Mmp9 | Matrix metallopeptidase 9 | 1.2025 | 0.7320 |
| Ncam1 | Neural cell adhesion molecule 1 | 0.8100 | 0.6029 |
| Nfkb1 | Nuclear factor of kappa light polypeptide gene enhancer in B-cells 1, p105 | 0.8444 | 0.8409 |
| Nfkbib | Nuclear factor of kappa light polypeptide gene enhancer in B-cells inhibitor, beta | 1.6888 | 1.0140 |
| Ngf | Nerve growth factor | 1.0762 | 0.6926 |
| Ngfr | Nerve growth factor receptor (TNFR superfamily, member 16) | 0.6764 | 0.6417 |
| Nos1 | Nitric oxide synthase 1, neuronal | 0.5456 | 0.5987 |
| Nptx2 | Neuronal pentraxin 2 | 0.8988 | 0.8236 |
| Nr4a1 | Nuclear receptor subfamily 4, group A, member 1 | 0.8044 | 0.8827 |
| Ntf3 | Neurotrophin 3 | 0.5889 | 0.5586 |
| Ntf5 | Neurotrophin 5 | 0.5532 | 0.3035 |
| Ntrk2 | Neurotrophic tyrosine kinase, receptor, type 2 | 0.7101 | 0.5743 |
| Pcdh8 | Protocadherin 8 | 1.1455 | 1.0000 |
| Pick1 | Protein interacting with C kinase 1 | 0.7505 | 0.7631 |
| Pim1 | Proviral integration site 1 | 1.1065 | 0.5905 |
| Plat | Plasminogen activator, tissue | 0.8444 | 0.6926 |
| Plcg1 | Phospholipase C, gamma 1 | 0.8503 | 0.8467 |
| Ppp1ca | Protein phosphatase 1, catalytic subunit, alpha isoform | 0.6718 | 0.7220 |
| Ppp1cc | Protein phosphatase 1, catalytic subunit, gamma isoform | 0.6954 | 0.6974 |
| Ppp1r14a | Protein phosphatase 1, regulatory (inhibitor) subunit 14A | 1.0837 | 0.8827 |
| Ppp2ca | Protein phosphatase 2 (formerly 2A), catalytic subunit, alpha isoform | 1.0614 | 0.6285 |
| Ppp3ca | Protein phosphatase 3, catalytic subunit, alpha isoform | 0.8503 | 0.6156 |
| Prkca | Protein kinase C, alpha | 0.7770 | 0.7170 |
| Prkcc | Protein kinase C, gamma | 0.8925 | 0.6690 |
| Prkg1 | Protein kinase, cGMP-dependent, type I | 0.7402 | 0.6113 |
| Rab3a | RAB3A, member RAS oncogene family | 0.6811 | 0.6242 |
| Rela | V-rel reticuloendotheliosis viral oncogene homolog A (avian) | 1.0112 | 0.8645 |
| Reln | Reelin | 0.6906 | 0.6974 |
| Rgs2 | Regulator of G-protein signaling 2 | 0.6764 | 0.8351 |
| Rheb | Ras homolog enriched in brain | 0.9240 | 0.8066 |
| Sirt1 | Sirtuin 1 (silent mating type information regulation 2, homolog) 1 (S. cerevisiae) | 0.6906 | 0.6199 |
| Srf | Serum response factor | 0.7770 | 0.7270 |
| Synpo | Synaptopodin | 0.8988 | 0.8950 |
| Timp1 | Tissue inhibitor of metalloproteinase 1 | 1.9399 | 1.2142 |
| Ywhaq | Tyrosine 3-monooxygenase/tryptophan 5-monooxygenase activation protein, theta polypeptide | 0.7402 | 0.5704 |
